# The Comparison of the Effect of Flour Particle Size and Content of Damaged Starch on Rice and Buckwheat Slurry, Dough, and Bread Characteristics

**DOI:** 10.3390/foods12132604

**Published:** 2023-07-05

**Authors:** Iva Burešová, Valérie Lullien-Pellerin, Libor Červenka, Jiří Mlček, Romana Šebestíková, Lucie Masaříková

**Affiliations:** 1 Department of Food Technology, Faculty of Technology, Tomas Bata University in Zlín, Nám. T. G. Masaryka 5555, 760 01 Zlín, Czech Republic; 2 INRAE, Institut Agro, IATE, University Montpellier, 2 place VIALA, Bât. 31, 34060 Montpellier, France; 3 Faculty of Chemical Technology, University of Pardubice, Studentská 95, 532 10 Pardubice, Czech Republic; 4 Department of Food Analysis and Chemistry, Faculty of Technology, Tomas Bata University in Zlín, Nám. T. G. Masaryka 5555, 760 01 Zlín, Czech Republic

**Keywords:** flour granulation, pasting properties, dough extensibility, bread crumb, bread quality

## Abstract

The effect of botanical origin, the flour particle size, and the content of damaged starch on flour pasting properties, dough behavior during a uniaxial deformation test, and bread characteristics were evaluated on rice and buckwheat flours. The rice flour with a median particle size D(0.5) of 60.2, 70.6, 106.8, and 189.4 μm, and buckwheat flour with a D(0.5) of 56.4, 68.4, and 95.8 μm were prepared using the same milling technology. The botanical origin of the flours was the strongest factor influencing the flour pasting properties, stress accumulated in dough during the uniaxial deformation test, loaf characteristics, texture, and sensory characteristics of breads. The flour particle size significantly influenced mainly the flour pasting properties. The effect of the content of damaged starch was the weakest among the studied factors. The flour particle size and the content of damaged starch were closely related. The flour botanical origin was the strongest factor; therefore, it seems not to be possible to predict the bread-baking potential of gluten-free flours based on the results obtained for flour of a different botanical origin. More research on flours from different plants prepared by the same milling process is required to support this hypothesis.

## 1. Introduction

The impact of flour particle size on flour, dough, and product quality has been studied by many authors. The studies were focused mainly on wheat flour, since it is often used in bread, pastry, cookies, and noodles. Wheat flour granulation was found to significantly impact flour properties [1,2].

The increasing demand for gluten-free products brought the need to also study the relation between flour particle size and bread quality in gluten-free flours. Some of the results obtained on wheat flour were found to also be applicable to rice and maize flour. Fine flour has higher damaged starch content [3,4] and exhibits a higher water-holding capacity and swelling volume than coarse flour [5,6]. The water hydration at a high temperature and pasting viscosity is rising, while the gelatinization temperature is decreasing with a reduction in the particle size [6]. Flour granulation also impacts rice and maize dough rheological characteristics. Dough from fine flours exhibits higher elastic modulus [5], stickiness, and limited expansion [7]. However, some results obtained by different authors vary. A high loaf-specific volume of bread prepared from fine rice flour was recorded by Qin et al. [6]. Luo et al. [7] found gluten-free bread prepared with medium-sized brown rice flour to have favorable quality characterized by large specific volume, low hardness, and numerous and homogeneous gas cells. Additionally, coarse maize flour was reported to be the most suitable for maize bread production [8]. It is evident that the results obtained by different authors may vary and cannot be easily generalized. This may be due to different flour milling and bread-baking procedures. Therefore, our study tried to overcome these weaknesses. The tested rice and buckwheat flours were prepared using the same milling procedure, as well as using the same bread-baking method.

Rice and buckwheat flours were involved in this study, since rice flour is one of the most used flours in the production of gluten-free bread. This flour exhibits several significant properties, such as bland taste, colorlessness, and hypoallergenic characteristics. A low level of protein, sodium, fat, and fiber and a high amount of easily digestible carbohydrates were also reported [9,10]. Buckwheat flour exhibited a higher potential to improve rice dough behavior and bread quality than amaranth, chickpea, corn, millet, and quinoa flours [11]. Moreover, it may be used to increase the nutritional quality of gluten-free bread. Buckwheat flour is a rich source of starch and contains many valuable compounds, such as proteins, antioxidative substances, unsaturated fatty acids, trace elements, and dietary fiber [12,13]. The low glycemic index of buckwheat food could be attributed to non-starch components. Buckwheat peptides have therapeutic potentials such as antiaging, modulation of gut microbiota, prevention of cardiovascular diseases, and a lowering of blood pressure. Immunomodulatory, antidiabetic, antimicrobial, anticancer, and antioxidant capacities were also reported [14]. The applicability of buckwheat flour in rice bread baking may also be supported by its price (3.38–6.34 EUR/1 kg), which is close to the price of rice flour (2.11–6.34 EUR/1 kg) in Czechia [15]. However, the prices may vary in different parts of the world. 

The aim of this study was to prepare rice and buckwheat flours of similar particle sizes using the same milling procedure. The pasting properties, dough behavior, and characteristics of bread prepared from rice and buckwheat flours were tested and compared. The effects of the flour botanical origin, particle size, and content of damaged starch on flour, dough, and bread parameters were evaluated, and their significance was compared. 

## 2. Materials and Methods

### 2.1. Flours

White rice (protein: 15 g; carbohydrates: 82 g; and fat: 1 g in 100 g of dry matter) and buckwheat seeds with a removed hull (protein: 17 g; carbohydrates: 76 g; and fat: 5 g in 100 g of dry matter) were bought in a local supermarket. Rice and buckwheat were in the form of a commercial blend. Information about the varieties used in the blends was not available. 

The same milling procedure was applied to the rice and buckwheat seeds. The seeds were grounded using a pin mill (FF Servis spol. s r.o., Prague, Czechia). The obtained flour was sifted to separate the flours with different granulations. The flours were vacuum-packed in polyethylene bags and stored at 4 °C before testing. The following sample abbreviations were used for rice flours: R60, R70, R100, and R200. For buckwheat flours, B60, B70, and B100 were used. The number in the flour names indicates the rounded-up value of the flour median particle size D(0.5).

### 2.2. Flour Characteristics

Flour particle size distribution was determined with light scattering using laser granulometry (Malvern Mastersizer 2000, Malvern Instruments SARL, Orsay, France), as described in Berton et al. [16], assuming a circular particle shape. For each sample, at least two independent replicates were analyzed. The different peaks were identified according to the obtained curves between volume and particle size. Integration of the obtained particle volume according to size was used to express the results as 10, 50, and 90% of the overall population. Monomodal particle size distribution was characterized by median D(0.5), D(0.9), D(0.1), and Span values. Median D(0.5) is the size in microns (µm) that splits the distribution in half above, and half below this value. Additionally, 90% of the distribution lies below the particle size given by D(0.9), and 10% lies below the particle size defined by D(0.1). Distribution width was also calculated and expressed as Span = [D(0.9) − D(0.1)]/D(0.5). Bimodal particle size distribution was characterized by particle size (µm) at the different maximum peak values and the percentage of particle volume in each of the peaks. 

The chemical composition of the flours was determined according to the ISO 1871, ISO 11085, and EN ISO 10520 [17,18,19] standards. Water absorption was determined using Mixolab 2 (Chopin Technologies, Villeneuve-la-Garenne, France). Water absorption was equal to the amount of demineralized water required to obtain a dough with the consistency of 1.10 ± 0.05 N m. Each test was performed on samples prepared with at least three replicates. The results are represented as mean values.

The level of damaged starch was determined using a Megazyme kit (K-SDAM starch damage assay kit, Megazyme Int., Wicklow, Ireland) according to the method AACCI N° 76-31.01 [20]. The results are mean values of two independent replicates with a standard deviation of <3%.

### 2.3. SEM Imaging

The flour samples were coated with a 2 nm layer of gold using a Q150R Plus Rotary Pumped Coater (Quorum, Laughton, UK) to improve their electrical conductivity. Images were taken by Tescan Vega3-SBU (Tescan Orsay Holding, a.s., Brno, Czechia) using backscattered electrons at the accelerating voltage of 10 kV and 800× magnification.

### 2.4. Flour Pasting Properties

HAAKE RheoStress 1 (Thermo Fisher Scientific Brno s.r.o., Brno, Czechia) was used for assessing the pasting properties of the flours. The suspension was prepared from flour (6.0 ± 0.1) g and water (30.0 ± 0.1) g. The rotation temperature ramp was performed using coaxial cylinders Z34 DIN Ti with a gap of 7.2 mm. The viscosity of samples was measured over a given period, during which the sample was being stirred. The profile of the test was: the addition of water to the flour sample, holding at the temperature of 30 °C for 120 s, heating to 90 °C for 220 s, holding at 90 °C for 300 s, cooling to 40 °C for 220 s and holding at 40 °C for 120 s. The suspension was stirred at 160 rpm during the test [21]. Each test was performed on samples prepared with at least three replicates. Slurry viscosity at 30 °C *η_30_*, pasting temperature *T_0_*, peak viscosity *η_Peak_*, final viscosity *η_Final_*, Breakdown, Total Setback, and Setback region were determined according to Balet et al. [22]. The results are represented as mean values.

### 2.5. Dough Behavior during Uniaxial Deformation

Dough samples were prepared according to the formula used in bread making (see Section 2.6), excluding yeast. The dough was prepared and tested according to the previously published method [23]. Texture analyzer TA.XT plus (Stable Micro System Ltd., Godalming, UK) equipped with an SMS/Kieffer Dough and Gluten Extensibility Rig was used to perform the uniaxial deformation test. During the test, the dough sample was stretched by the hook until it fractured. The hook speed during the test was 3.00 mm/s, and the trigger force was 5 g. The obtained values were recalculated into stress–strain curves as described by Dunnewind et al. [24]. The value of peak stress *σ_M_* and peak Hencky strain *ε_HM_* were used to describe dough behavior. Each test was performed on dough samples prepared in at least six replicates. The given results are represented as mean values.

### 2.6. Bread Preparation

Breads were prepared according to the previously published method [25]. The dough was prepared from flour (100 g), water (90 g), sucrose (1.86 g), salt (1.00 g), and dry yeast (1.80 g); the amounts of ingredients were related to flour dry matter. The ingredients were kneaded for (6 ± 1) min in an Eta Gratus mixer bowl (ETA a.s., Prague, Czechia). The prepared dough (600 g) was scaled into bread pans of 9.4 × 18.3 × 7.0 cm and placed into a proofer for (20 ± 2) min at (30 ± 1) °C and 85% relative air humidity. The loaves were baked for (40 ± 2) min at (180 ± 5) °C (MIWE cube, Pekass s.r.o., Plzeň, Czechia). The baked loaves were removed from the pans and stored at room temperature (21 ± 3) °C for 2 h. Loaf volume was determined using plastic granulates. Loaf-specific volume (mL/g) was calculated by dividing the bread volume by bread weight. Three batches of three varieties of bread were baked for each flour. The results are represented as mean values.

Texture profile analysis (TPA) of bread crumbs was performed on TA.XT plus texture analyzer (Stable Micro Systems Ltd., UK) according to a previously published method [25]. A 75.0 mm diameter cylinder probe P/75 was used. The parameters of the test were: pre-test speed 1.00 mm/s; test speed 5.00 mm/s, strain 40%, and trigger force 5 g. Crumb hardness, springiness, cohesiveness, resilience, and chewiness were calculated according to Trinh and Glasgow [26]. At least three samples obtained from each bread were tested. The results are represented as mean values.

The sensory attributes of bread were evaluated by 5 panelists trained according to ISO 8586-1 [27]. The panelists were both male and female, aged 26–52 years (the department staff and students). The sensory evaluation was performed under standard conditions (ISO 8589) [28]. An unstructured 10 cm long scale was used to evaluate the characteristics of bread crumbs and crust [29]. The attribute of intensity/acceptability increased from left to right. The results are represented as mean values.

### 2.7. Statistical Analysis

The results were statistically analyzed using analysis of variance (ANOVA). The differences between samples were tested on a 0.05 significance level using the Tukey test. Parameter dependency was tested using a Wilcoxon test and *t*-test on a 0.05 level. The statistical analyses were performed using Statistica 13.0 (TIBCO Software s.r.o., Prague, Czechia).

## 3. Results and Discussion

### 3.1. Flour Characteristics

**Figure 1:**
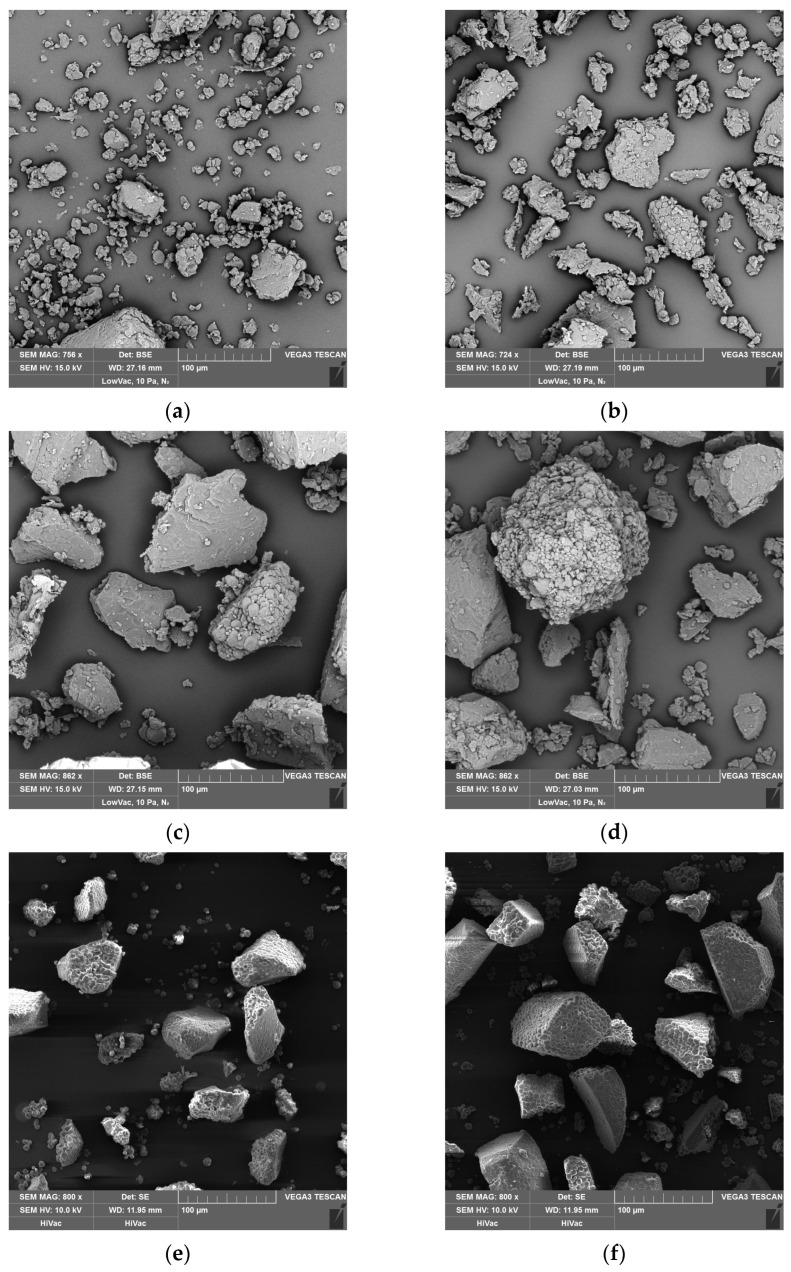

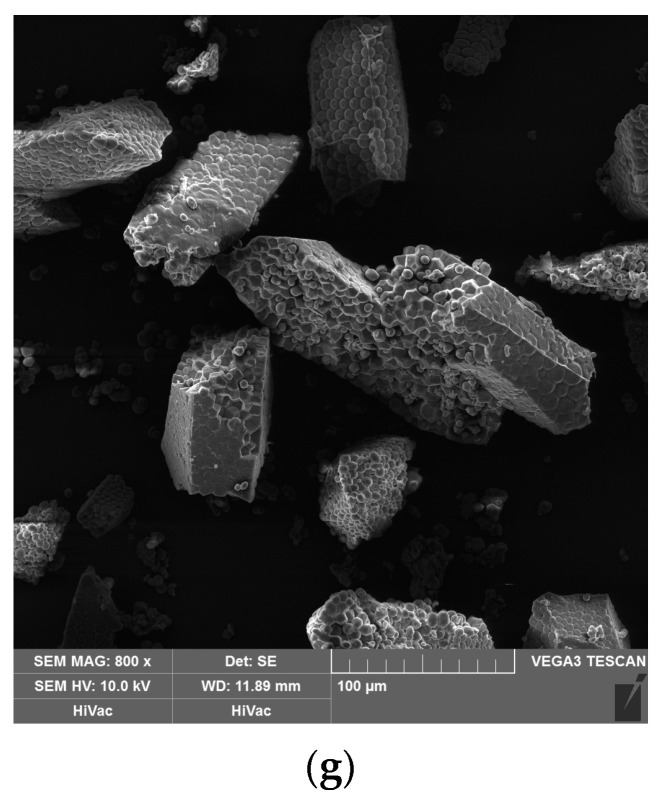
SEM of rice flours. (**a**) R60: detached starch granules, small fragments of endosperm, several pieces of larger-sized fragments of endosperm; (**b**) R70: fragments of endosperm with a median particle size of 70.64 µm; (**c**) R100: fragments of endosperm with a median particle size of 106.78 µm; (**d**) R200: a mixture of smaller- and larger-sized fragments of endosperm; (**e**) B60: detached starch granules, particles created by several starch granules joined together, fragments of endosperm with a mean size of 79.62 µm; (**f**) B70: detached starch granules, particles created by several starch granules joined together, fragments of endosperm with a mean size of 89.34 µm; (**g**) B100: detached starch granules, particles created by several starch granules joined together, fragments of endosperm with a mean size of 100.24 µm. The number in the flour names indicates the rounded-up value of flour median particle size D(0.5).

Four rice flours (R60, R70, R100, and R200) and three buckwheat flours (B60, B70, and B100) were collected during the milling process (Table 1). The portion of particles separated as buckwheat flour B200 was not sufficient for all intended tests; therefore, this flour was excluded from this study. The differences in the effectiveness of grinding may be related to the variability in the hardness of rice and buckwheat seeds [4]. The different behavior during the milling process is also evident from particle size distribution. The distribution was monomodal in rice flours. Two peaks were detected in each tested buckwheat flour. These flours exhibited a bimodal particle distribution. The first peak was created in all buckwheat flours by small particles of similar size (10.02–11.25 µm). Some starch granules loosened during flour milling, and several starch granules joined together, a phenomenon present in all tested buckwheat flours (Figure 1e–g). These particles were mainly present in the first peaks. The size of the particles present in the second peaks varied between 79.6 µm and 100.2 µm (Table 1).

The content of starch was higher in fine flours (Table 2). The content of protein exhibited the opposite trend. The starch, protein, and other substances were not homogenously distributed in seeds. The particles present in fine flours were probably situated mainly in the softer parts of the seeds. These parts are known to disintegrate into smaller particles [30], which were collected in fine flours. The harder parts of the endosperm disintegrated into large particles collected in coarse flours. However, the coarse rice flour R100 did not follow this trend. A similar observation was published by de la Hera et al. [31]. 

The content of damaged starch varied from 5.9% to 13.3% in the rice flours and from 1.2% to 3.4% in the buckwheat flours (Table 2). This parameter was significantly lower in the buckwheat flours than in the rice flours with a similar median particle size, which is in general agreement with the results reported by Torbica et al. [32]. Fine flours generally had a higher content of damaged starch than coarse flours. A similar relation between flour granulation and the content of damaged starch has already been previously described [1,5,33]. Moreover, the content of damaged starch and flour particle size was significantly dependent on each other (*p* < 0.05). The level of damaged starch was probably impacted by the different behavior of buckwheat and rice grains during grinding. During the milling process, seeds were subjected to various forces, making them break up into smaller particles. A certain portion of starch granules were loosened from the protein matrix and remained undamaged. During rice milling, the granules were separated mainly in R60 flour (Figure 1a). They were not present in other rice flours (Figure 1b–d). They had a diluting effect on the content of damaged starch in this flour. This hypothesis may be supported by the values of water absorption. Water absorption is known to be increased by the presence of damaged starch [4]. This parameter was higher in R70 than in R60. The diluting effect of the loosened starch granules was not observed in buckwheat flours, since these granules were present in all tested buckwheat flours (Figure 1e–g). However, their amount decreased with increasing flour particle size.

Water absorption was influenced by flour botanical origin (*p* = 0.13). The effect of the content of damaged starch was weak (*p* = 0.57) and was observed mainly in rice flours. A wider difference in the content of damaged starch in rice flours than in buckwheat flours may be a possible explanation.

### 3.2. Flour Pasting Properties

Slurry viscosity at the initial phase of the test *η_30_* was significantly influenced by flour botanical origin (*p* = 0.007), the content of damaged starch (*p* = 0.001), and flour particle size (*p* = 0.001). The slurries with buckwheat flours exhibited significantly higher viscosity *η_30_* than slurries containing rice flours (Table 3). Bimodal particle size distribution in buckwheat flours, mainly the presence of coarse particles (detected as second peak), decreased slurry viscosity, since water slowly penetrated the inner parts of these flour particles and a part of water remained unbound, decreasing slurry viscosity. A similar observation was also previously described for wheat flour [33]. The viscosity at this phase of the test is impacted by the hydration of the substances present in flour [22]. Moreover, damaged starch granules are known to be able to absorb a large amount of water [4]. However, the relation between the content of damaged starch and viscosity *η_30_* was only valid in flours of the same botanical origin. Even if a higher content of damaged starch was observed in rice flours, the viscosity of slurries with these flours was lower than the viscosity of slurries with buckwheat flours with a lower level of damaged starch. Therefore, the combination of different content of the water-binding ability of the substances (proteins, starch) in rice and buckwheat flours (Table 2), and the differences in their characteristics, is a possible explanation.

The effect of flour botanical origin was significant during the heating phase of the test, but less so (*p* = 0.019) during the initial phase. The effect of damaged starch was also significant (*p* = 0.027). The viscosity started to rise when the temperature *T*_0_ of the slurry reached (50–66) °C. Peak viscosity *η_Peak_* varied between (91 and 472) mPa·s and final viscosity *η_Final_* between (163 and 540) mPa·s. The differences between slurries with rice and buckwheat flours observed in *η*_0_ values disappeared during the heating phase of the test, which may be related to a more rapid increase of viscosity recorded in the slurry with rice flours. Temperature *T*_0_, peak viscosity, and final viscosity generally declined with increasing content of damaged starch. According to Barrera, gelatinized damaged granules are probably broken more easily and are more deformable than less damaged granules. Therefore, damaged starch granules do not fully contribute to viscosity increment during slurry heating [34], decreasing the viscosity of slurries with fine flours.

The breakdown was significantly impacted by flour particle size (*p* = 0.001). This parameter generally rose with increasing median particle size. A similar relation was observed by Ma et al. [2]. Total setback and Setback region were significantly influenced by botanical origin and flour particle size, as well as the content of damaged starch (*p* = 0.001). These parameters were higher in slurries with buckwheat flours and in slurries with fine flours. The setback region decreased with increasing flour particle size, which corresponds to previously published results [6], indicating a low tendency of coarse flour to retrograde [22]. 

Pasting properties recorded in the slurry with buckwheat B70 flour differed from the described trends. This slurry exhibited the highest viscosity during the initial phase of the test *η*_30_ among all tested flours. This flour exhibited a lower size of particles present in the first peak than B60 and B100 flours. The content of damaged starch is higher in fine particles; therefore, the level of damaged starch was probably higher in these particles than in the particles present in the first peaks of B60 and B100 flours. Additionally, damaged starch granules are known to be able to absorb large amounts of water [4], increasing slurry viscosity. This explanation is supported by the value of water absorption, which was higher in B70 than in B60 and B100 flours. During the heating phase of the test, the differences in viscosities *η_Peak_* and *η_Final_* recorded in slurries with B60, B70, and B100 were not as large as in *η*_30_. Slurry viscosity was probably influenced mainly by the amount and characteristics of coarse flour particles present in second peaks, since the content of damaged starch decreased with increasing particle size.

### 3.3. Behavior of Dough during the Uniaxial Elongation Test

Dough behavior during the uniaxial deformation test was impacted by botanical origin (*p* = 0.014), flour particle size (*p* = 0.001), and the content of damaged starch (*p* = 0.006). The comparison of the flours of similar median particle size indicates that rice doughs exhibited a generally higher ability to accumulate stress during the uniaxial elongation test than buckwheat doughs (Table 4). Dough behavior during the test is known to be influenced by the quality and quantity of flour components, especially proteins, and starch. The differences in rice and buckwheat protein quality and quantity may contribute to the differences in dough behavior. Additionally, dough behavior is impacted by the loss of adhesion between protein and starch [35,36]. Damaged starch granules probably decreased adhesion, resulting in a weaker dough with a low ability to accumulate stress. This hypothesis may be supported by the results of the dough prepared from R70 flour. This flour exhibited a high content of damaged starch (13.3%). Its ability to accumulate stress was also the lowest among the tested rice flours. 

Botanical origin was another factor influencing dough behavior. Rice dough exhibited higher values of Peak stress *σ_M_* and Hencky peak strain *ε_HM_*, which may be related to differences in the characteristics of rice and buckwheat protein, starch, and other substances. 

Rice dough’s ability to accumulate stress *σ_M_* rose with rising flour median particle size. A similar relation was observed in relative dough deformation expressed as Hencky peak strain *ε_HM_*; however, the significance of the differences was weak. Flour particles disintegrate during dough mixing. This process is more rapid in smaller-sized particles. Smaller-sized particles probably completely disintegrated at the end of dough mixing. A part of larger-sized particles remained in the dough, strengthening it, which was observed as higher values of Hencky stress *σ_M_* and Hencky peak strain *ε_HM_*. The tested buckwheat doughs did not exhibit any clear trend. Bimodal particle size distribution in buckwheat flours may impact dough behavior, since the homogeneity of particle distribution impacts the dough behavior during the large-scale deformation test and the fracture stress [35,37]. 

### 3.4. Bread Quality

All tested factors impacted bread characteristics. The impact of botanical origin was recorded on loaf-specific volume, crumb hardness, chewiness, and springiness (*p* = 0.001). The effect on baking loss (*p* = 0.002) and crumb cohesiveness (*p* = 0.046) was weaker. Buckwheat bread exhibited higher loaf-specific volume, springiness, cohesiveness, and chewiness than bread made from rice flours (Table 5). The better quality of buckwheat breads may be related to the characteristics of proteins, starch, and their behavior during baking, which was also observed during the heating test. Buckwheat flours seemed to have better bread-making potential than rice flours.

Flour particle size significantly influenced crumb hardness (*p* = 0.001), chewiness (*p* = 0.010), and springiness (*p* = 0.020). These parameters rose with the increasing particle size in the rice flours, which had a detrimental impact on bread quality. A similar negative effect of coarse rice flour on breadcrumb hardness was previously observed by Qin et al. [6]. The relation between flour particle size and crumb characteristics was not clear in buckwheat flours. The bread prepared from B100 exhibited better characteristics than bread prepared from finer buckwheat flours. This may indicate the importance of particle size distribution. It may be hypothesized that the presence of coarser flour particles in buckwheat flour, detected as the second peak, had a positive effect on bread characteristics, like those recorded by de la Hera et al. [8,38]. Additionally, Belorio et al. [5] reported that a certain percentage of fine particles placed between coarser particles in the dough might positively influence some parameters of the quality of the product. 

The content of damaged starch significantly impacted loaf-specific volume (*p* = 0.007) and baking loss (0.032). Both parameters rose with the decreasing level of damaged starch. High loaf volume is associated with high-quality bread. The effect of damaged starch on loaf volume may be expected due to a known impact of damaged starch on dough viscosity. Optimal viscosity is necessary to trap leavening gas in the dough. Damaged granules gelatinized during baking were probably broken more easily and did not fully contribute to viscosity increment during heating [34], decreasing the dough’s ability to trap leavening gas. The baking loss also rose with the decreasing content of damaged starch. The value of baking loss is associated with water evaporation during baking. Extensive water evaporation may result in a dry and hard bread crumb.

**Figure 2:**
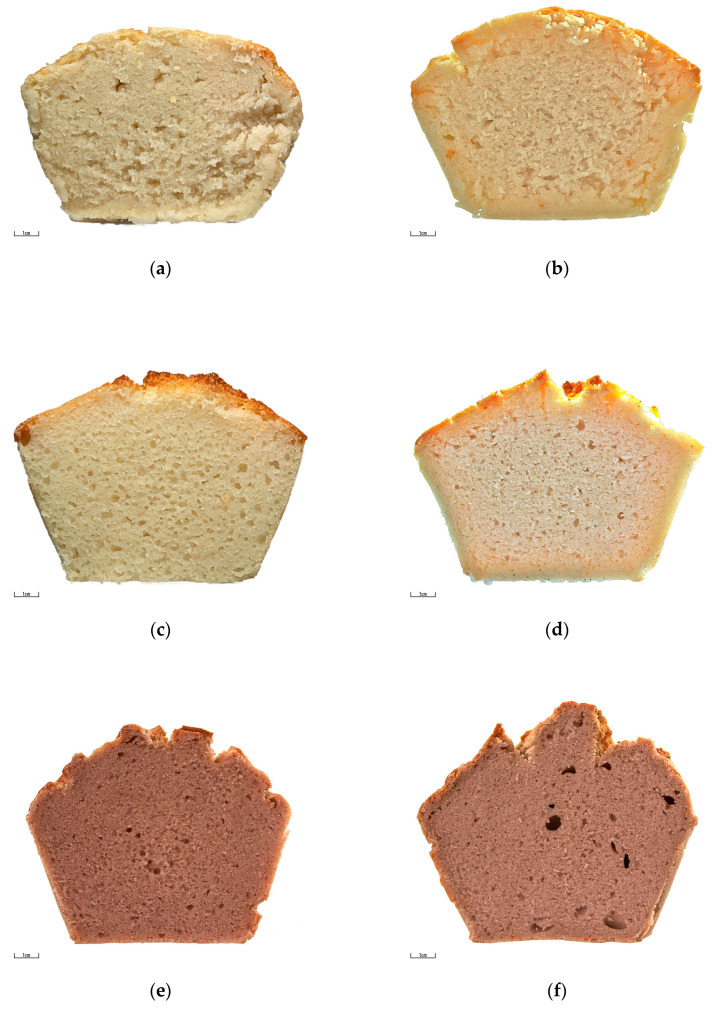

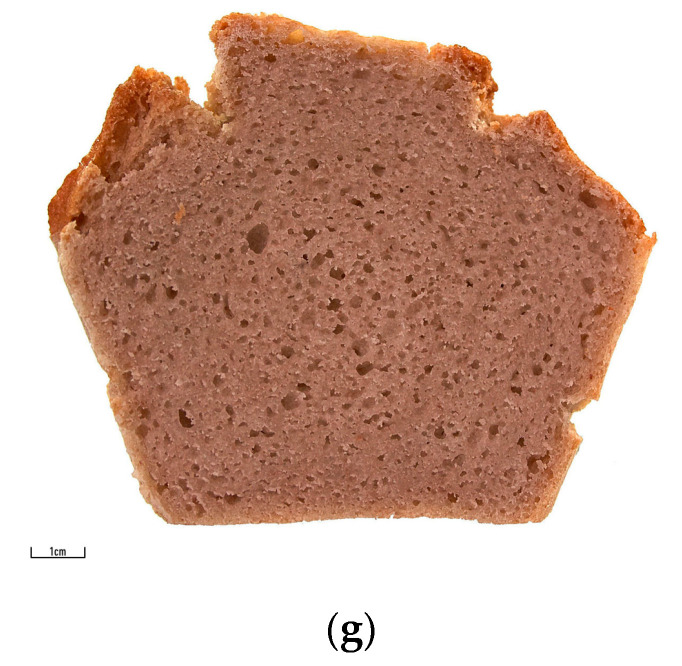
The crumb of bread prepared from flours of different botanical origins and particle sizes. (**a**) small-sized pores in partly open crumbs in bread from rice R60 flour; (**b**) small-sized pores in partly open crumbs in bread from rice R70 flour; (**c**) homogenously distributed larger-sized pores in partly open crumbs in bread from rice R100 flour; (**d**) irregularly distributed larger-sized pores in open crumb in bread from rice R200; (**e**) small enclosed pores in bread from buckwheat B60 flour; (**f**) slightly larger-sized enclosed pores in bread from buckwheat B70 flour; (**g**) open crumb structure with large pores in bread from buckwheat B100 flour.

The results of the sensory evaluation are presented in Table 6. Sensory attributes of bread (crust color, crust uniformity, crumb color, crumb hardness, uniformity of pores, flavor, odor, and overall acceptability) were significantly impacted by flour botanical origin. The different color of rice and buckwheat bread is evident in Figure 2. Plain color is typical for rice bread. The darker color of buckwheat bread is caused by the presence of polyphenols in buckwheat seeds [39,40]. Even if the instrumental determination of bread color was not involved in our study, the color characteristics of rice and buckwheat bread were reported by Baldino et al. [41] and Coronel et al. [42]. The crumb of rice bread tested by Baldino et al. [41] was characterized by L* = 78; a* = 2.3; b* = 11.8, and the crumb of buckwheat bread prepared by Coronel et al. [42] was characterized by L* = 55.58; a* = 4.91; b* = 12.35. The darker color of buckwheat bread was favored by panelists. The difference in the color evaluation was most evident in crumb color. The differences in crust color were not so extensive due to the crust browning during baking.

Crumb hardness, size of pores, and uniformity of pores are closely related parameters. Crumb hardness and size of pores were evaluated more positively in bread prepared from buckwheat flours. However, the distribution of pores was more homogeneous in rice bread. This parameter was evaluated more negatively, mainly due to several larger-sized pores that were irregularly distributed in the crumb (Figure 2a,e,f). The overall acceptability of bread was significantly impacted by bread flavor and odor. Flavor and odor were more prominent in buckwheat bread compared to rice bread. The panelists preferred the plain flavor and odor, which resulted in rice bread being evaluated more positively.

Flour particle size significantly influenced the size of pores (*p* = 0.001). Larger-sized pores and homogeneous porosity were observed in bread from the R100 and B100 flours (Figure 2c,g). The effect of the content of damaged starch on sensory attributes was not significant.

## 4. Conclusions

The botanical origin of the flours was the strongest factor influencing flour pasting properties, dough behavior during the uniaxial deformation test, and bread characteristics. Pasting properties were impacted by botanical origin mainly during the initial phase of the test and during the cooling stage. During the uniaxial deformation test, the tested rice doughs exhibited a slightly better behavior than the doughs from buckwheat flours. The effect of botanical origin on bread characteristics (loaf-specific volume, crumb hardness, chewiness, and springiness) was significant. Flour particle size mainly influenced the pasting properties of the flour. The effect of the content of damaged starch was the weakest among the studied factors. Moreover, flour particle size and the content of damaged starch were closely related.

The sensory attributes of bread were strongly influenced by flour’s botanical origin. The effect of the other factors was marginal. Sensory characteristics determine if the bread is acceptable to consumers. Therefore, flour botanical origin is the strongest and most important factor influencing the bread-making potential of gluten-free flours. Moreover, it seems to not be possible to predict the bread baking potential of gluten-free flours based on the results obtained for flours of different botanical origins. More research on flours from different plants prepared by the same milling process is required to support this hypothesis.

## Figures and Tables

**Table 1:** Flour granule size distribution: Type of distribution; median particle size D(0.5), values of D(0.1) and D(0.9); the size of particles (µm) detected as 1st, 2nd peak; the percentage of particle volume in each of the peaks; content of damaged starch ^1^.

Flour	Distribution of Flour Particles	D(0.50) μm	D(0.10) μm	D(0.90) μm	SPAN	1st Peak μm	Particle Volume %	2nd Peak μm	Particle Volume %
R60	Monomodal	60.2	10.5	148.1	2.28	-	-	-	-
R70	Monomodal	70.6	8.7	208.9	2.83	-	-	-	-
R100	Monomodal	106.8	19.7	251.1	2.17	-	-	-	-
R200	Monomodal	189.4	36.7	451.2	2.19	-	-	-	-
B60	Bimodal	56.4	9.7	141.6	2.34	11.25	28.7	79.62	71.3
B70	Bimodal	68.4	7.1	159.0	2.22	10.02	22.8	89.34	77.2
B100	Bimodal	95.8	39.9	267.0	2.37	11.25	7.2	100.24	92.8

^1^ R: rice; B: buckwheat. The number in the flour names indicates the rounded-up value of flour median particle size D(0.5).

**Table 2:** Flour characteristics ^2^. Water absorption, content of protein, fat, carbohydrates, and damaged starch (%).

Flour	Protein	Fat	Carbohydrates	Damaged Starch	Water Absorption
R60	6.0 ± 0.2 cd	1.2 ± 0.2 c	92.8 ± 0.6 a	10.8	69.45 ± 0.07 b
R70	6.8 ± 0.9 c	0.8 ± 0.2 cd	92.4 ± 0.8 a	13.3	70.05 ± 0.07 a
R100	6.9 ± 0.3 c	1.0 ± 0.2 cd	90.1 ± 0.5 b	5.4	68.35 ± 0.08 e
R200	6.1 ± 0.8 cd	0.7 ± 0.3 d	93.2 ± 0.3 a	5.9	68.43 ± 0.04 de
B60	5.8 ± 0.2 d	1.9 ± 0.3 b	92.3 ± 0.3 a	3.4	68.56 ± 0.04 cd
B70	8.3 ± 0.6 b	1.7 ± 0.2 b	90.0 ± 0.4 b	3.0	68.69 ± 0.02 c
B100	12.0 ± 0.8 a	2.9 ± 0.6 a	85.1 ± 0.4 c	1.2	68.62 ± 0.02 c

^2^ R: rice; B: buckwheat. The number in the flour names indicates the rounded-up value of flour median particle size D(0.5). The mean values (*n* = 3) followed by different letters in the column differ significantly (*p* < 0.05).

**Table 3:** Pasting properties of the tested rice and buckwheat flours ^3^.

Flour	*η*_30_ (mPa·s)	*T*_0_ (°C)	*η_Peak_* (mPa·s)	*η_Final_* (mPa·s)	Breakdown (mPa·s)	Total Setback (mPa·s)	Setback Region (mPa·s)
R60	3.1 ± 0.2 d	51 ± 7 c	91 ± 10 d	163 ± 7 c	27 ± 4 c	92 ± 13 b	79 ± 12 c
R70	3.2 ± 0.3 d	56 ± 9 bc	85 ± 10 d	80 ± 10 d	78 ± 9 b	90 ± 10 b	12 ± 10 e
R100	2.6 ± 0.2 e	54 ± 9 c	453 ± 10 a	510 ± 16 a	63 ± 9 b	107 ± 40 b	44 ± 12 d
R200	2.5 ± 0.2 e	63 ± 2 b	472 ± 8 a	540 ± 30 a	18 ± 5 cd	69 ± 33 b	51 ± 17 d
B60	16.0 ± 0.9 b	58 ± 6 bc	112 ± 10 c	360 ± 10 b	18 ± 4 d	262 ± 12 a	244 ± 15 a
B70	72.9 ± 0.8 a	50 ± 3 c	196 ± 30 b	380 ± 30 b	24 ± 5 c	204 ± 32 a	180 ± 29 b
B100	6.3 ± 0.2 c	66 ± 3 a	115 ± 10 c	390 ± 40 b	92 ± 1 a	276 ± 60 a	184 ± 16 b

^3^ R: rice; B: buckwheat. The number in the flour names indicates the rounded-up value of flour median particle size D(0.5). *η*_30_: slurry viscosity at 30 °C, *T*_0_: pasting temperature, *η_Peak_*: peak viscosity, *η_Final_*: final viscosity, Breakdown, Total Setback, and Setback region. Mean values (*n* = 3) followed by different letters in the column differ significantly (*p* < 0.05).

**Table 4:** Dough behavior under uniaxial deformation. Peak stress *σ_M_* and Hencky strain *ε_HM_* at the moment of dough rupture ^4^.

Flour	Peak Stress *σ_M_* kPa	Hencky Peak Strain *ε_HM_* -
R60	4.4 ± 0.5 c	0.58 ± 0.02 c
R70	3.8 ± 0.3 cd	0.73 ± 0.02 a
R100	5.9 ± 0.3 b	0.74 ± 0.02 a
R200	9.7 ± 0.9 a	0.77 ± 0.03 a
B60	3.7 ± 0.5 d	0.71 ± 0.02 b
B70	2.1 ± 0.2 e	0.67 ± 0.05 b
B100	4.4 ± 0.2 c	0.73 ± 0.02 a

^4^ R: rice; B: buckwheat. The number in the flour name indicates the rounded-up value of flour median particle size D(0.5). The mean values ± standard deviation (*n* = 6) followed by different letters in the column differ significantly (*p* < 0.05).

**Table 5:** Loaf-specific volume, baking loss, and the characteristics of bread crumbs measured by TPA ^5^.

Flour	Loaf Specific Volume mL/g	Baking Loss %	Hardness N	Springiness %	Cohesiveness %	Resilience %	Chewiness J
R60	1.24 ± 0.02 e	16.6 ± 0.2 a	28 ± 2 e	74 ± 8 cd	74 ± 6 cd	49 ± 3 abc	15.3 ± 1.2 d
R70	1.02 ± 0.02 g	13.2 ± 0.2 cd	55 ± 2 b	75 ± 8 cd	82 ± 4 ab	51 ± 4 ab	34.0 ± 1.9 c
R100	1.27 ± 0.02 d	17.5 ± 0.2 a	48 ± 3 cd	85 ± 3 bc	81 ± 1 b	49 ± 1 abc	33.0 ± 1.7 d
R200	1.29 ± 0.04 cd	15.9 ± 0.2 ab	70 ± 3 a	91 ± 3 ab	77 ± 3 c	47 ± 3 abc	49.7 ± 2.8 a
B60	1.38 ± 0.02 b	15.9 ± 0.6 ab	51 ± 1 bc	85 ± 4 bc	87 ± 8 ab	46 ± 3 abc	37.7 ± 2.9 bc
B70	1.31 ± 0.02 c	13.8 ± 0.6 bcd	57 ± 2 b	93 ± 6 ab	82 ± 9 ab	48 ± 4 abc	43.4 ± 4.4 ab
B100	1.43 ± 0.03 a	14.6 ± 0.9 bc	46 ± 2 d	95 ± 2 a	86 ± 5 a	53 ± 1 a	37.4 ± 1.4 b

^5^ R: rice; B: buckwheat. The number in flour names indicates the value of flour median particle size D(0.5), rounded up. Mean values ± standard deviation (*n* = 9), followed by different letters in the column, differ significantly *p* < 0.05.

**Table 6:** Sensory analysis of bread ^6^.

Flour	Crust Color	Crust Uniformity	Crumb Color	Size of Pores	Uniformity of Pores	Crumb Hardness	Flavor Intensity	Flavor Aftertaste	Odor Intensity	Overall Acceptability
R60	5.3 ± 0.6 c	1.0 ± 0.7 d	1.9 ± 0.5 b	3.6 ± 0.6 d	5.1 ± 0.2 a	7.7 ± 0.2 bc	2.2 ± 0.3 b	1.1 ± 0.2 a	4.4 ± 0.6 b	9.3 ± 0.4 a
R70	3.2 ± 0.3 d	7.2 ± 0.5 b	1.8 ± 0.7 b	5.0 ± 0.7 cd	5.4 ± 0.3 a	1.1 ± 0.2 e	1.8 ± 0.4 b	1.3 ± 0.4 a	4.3 ± 0.4 b	6.2 ± 0.2 b
R100	2.3 ± 0.4 d	9.3 ± 0.2 a	2.2 ± 0.6 b	8.6 ± 0.6 a	5.4 ± 0.6 a	6.7 ± 0.5 c	1.5 ± 0.2 b	1.2 ± 0.2 a	4.6 ± 0.2 b	6.7 ± 0.2 b
R200	3.1 ± 0.2 d	4.3 ± 0.3 c	2.4 ± 0.6 b	5.0 ± 0.7 cd	5.3 ± 0.2 a	2.8 ± 0.3 d	2.1 ± 0.4 b	1.3 ± 0.4 a	4.9 ± 0.2 b	6.3 ± 0.2 b
B60	5.5 ± 0.2 c	5.4 ± 0.6 bc	8.3 ± 0.7 a	6.1 ± 0.2 bc	3.2 ± 0.7 b	8.1 ± 0.2 ab	8.9 ± 0.4 a	1.0 ± 0.2 a	8.9 ± 0.4 a	4.3 ± 0.2 c
B70	7.1 ± 0.7 b	4.5 ± 0.4 c	8.5 ± 0.4 a	6.2 ± 0.2 bc	3.0 ± 0.2 b	8.1 ± 0.2 ab	9.3 ± 0.2 a	1.4 ± 0.2 a	9.3 ± 0.2 a	4.1 ± 0.2 c
B100	8.8 ± 0.4 a	5.7 ± 0.5 bc	6.9 ± 0.2 a	7.1 ± 0.2 ab	4.1 ± 0.2 ab	8.9 ± 0.2 a	9.2 ± 0.4 a	1.2 ± 0.2 a	9.2 ± 0.4 a	4.5 ± 0.2 c

^6^ R: rice; B: buckwheat. The number in the flour names indicates the value of flour median particle size D(0.5), rounded up. The mean values ± standard deviation (*n* = 5) followed by different letters in the column differ significantly *p* < 0.05.

## Data Availability

Results are available from the corresponding author.
